# The syndrome of transient epileptic amnesia: a combined series of 115 cases and literature review

**DOI:** 10.1093/braincomms/fcab038

**Published:** 2021-03-13

**Authors:** John Baker, Sharon Savage, Fraser Milton, Christopher Butler, Narinder Kapur, John Hodges, Adam Zeman

**Affiliations:** 1 Cognitive & Behavioural Neurology, University of Exeter Medical School, College House, St Luke’s Campus, Exeter EX1 2LU, UK; 2 School of Psychology, University of Newcastle, New South Wales 2308, Australia; 3 Discipline of Psychology, University of Exeter, Washington Singer Laboratories, Exeter EX4 4QG, UK; 4 Nuffield Department of Clinical Neurosciences, University of Oxford, John Radcliffe Hospital, Oxford OX3 9DU, UK; 5 Department of Brain Sciences, Imperial College, London W12 0NN, UK; 6 Departamento de Neurología, Pontificia Universidad Católica de Chile, Santiago 833007, Chile; 7 Research Department of Clinical, Educational and Health Psychology, University College London, London WC1E 6BT, UK; 8 Brain and Mind Centre, University of Sydney, Sydney 2050, Australia

**Keywords:** epilepsy, memory, neuropsychology, autobiographical amnesia

## Abstract

The term transient epileptic amnesia was coined in 1990 to describe a form of epilepsy causing predominantly amnestic seizures which could be confused with episodes of Transient Global Amnesia. Subsequent descriptions have highlighted its association with ‘atypical’ forms of memory disturbance including accelerated long-term forgetting, disproportionate autobiographical amnesia and topographical amnesia. However, this highly treatment-responsive condition remains under-recognized and undertreated. We describe the clinical and neuropsychological features in 65 consecutive cases of transient epileptic amnesia referred to our study, comparing these to our previous cohort of 50 patients and to those reported in 102 literature cases described since our 2008 review. Findings in our two cohorts are substantially consistent: The onset of transient epileptic amnesia occurs at an average age of 62 years, giving rise to amnestic episodes at a frequency of around 1/month, typically lasting 15–30 min and often occurring on waking. Amnesia is the only manifestation of epilepsy in 24% of patients; olfactory hallucinations occur in 43%, motor automatisms in 41%, brief unresponsiveness in 39%. The majority of patients describe at least one of the atypical forms of memory disturbance mentioned above; easily provoked tearfulness is a common accompanying feature. There is a male predominance (85:30). Epileptiform changes were present in 35% of cases, while suspected causative magnetic resonance imaging abnormalities were detected in only 5%. Seizures ceased with anticonvulsant treatment in 93% of cases. Some clinical features were detected more commonly in the second series than the first, probably as a result of heightened awareness. Neuropsychological testing and comparison to two age and IQ-matched control groups (*n* = 24 and 22) revealed consistent findings across the two cohorts, namely elevated mean IQ, preserved executive function, mild impairment at the group level on standard measures of memory, with additional evidence for accelerated long-term forgetting and autobiographical amnesia, particularly affecting episodic recollection. Review of the literature cases revealed broadly consistent features except that topographical amnesia, olfactory hallucinations and emotionality have been reported rarely to date by other researchers. We conclude that transient epileptic amnesia is a distinctive syndrome of late-onset limbic epilepsy of unknown cause, typically occurring in late middle age. It is an important, treatable cause of memory loss in older people, often mistaken for dementia, cerebrovascular disease and functional amnesia. Its aetiology, the monthly occurrence of seizures in some patients and the mechanisms and interrelationships of the interictal features—amnestic and affective—all warrant further study.

## Teaser

Transient epileptic amnesia is a distinctive syndrome of late-onset limbic epilepsy occurring in late middle age and is a treatable cause of memory loss in older people. Here, Baker et al. describe 65 cases, comparing these to their previous cohort of 50 patients and to 102 literature cases.

## Graphical Abstract

**Graphical Abstract:**
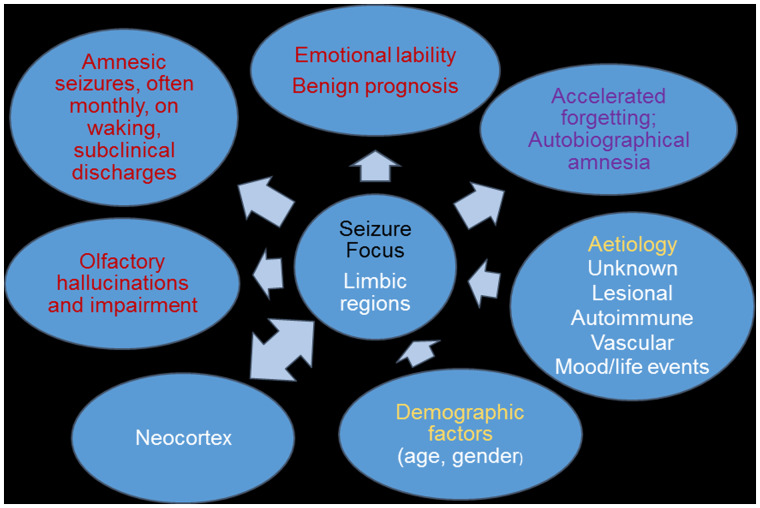

## Introduction

The term ‘transient epileptic amnesia’ (TEA) was coined in 1990 to highlight the existence of a distinctive form of epilepsy causing transient amnesic attacks.^1^^,^^2^ Their superficial resemblance to the attacks occurring in ‘transient global amnesia’ (TGA) warranted a related but contrasting term. Hughlings-Jackson^3^ was probably the first author to raise the possibility that transient amnesia could be the sole or most prominent manifestation of an epileptic seizure, in his description of his physician–patient, Dr Z. The suggestion was supported by case reports over the following century, preceding the definition of TEA.^4^ Since then, further reports have defined an epilepsy syndrome characterized by recurrent brief attacks of transient amnesia, often occurring on waking, with onset typically in middle age.^5–7^

The syndrome is of particular neuropsychological interest as the amnestic seizures are frequently accompanied by a distinctive group of persistent interictal memory complaints: accelerated long-term forgetting (ALF), autobiographical amnesia (AbA) and topographical amnesia (TopA).^4^^,^^6^^,^^8^ ALF is the excessively rapid loss of memory, over extended intervals, of information that appears to have been acquired and stored normally over standard testing intervals of around half an hour. AbA refers to the loss of memories for all or part of one’s past life: This often comes to light when reviewing family photographs or reminiscing with friends and relations, and particularly affects the rich, ‘experiential’ or ‘autonoetic’ recall of salient personal events. TopA involves difficulty in recollecting the layout of previously familiar environments, often when driving, and/or a failure to recognize previously familiar landmarks and locations. While these measurable memory problems have been described as features in other types of epilepsy, and in other clinical contexts, they occur particularly commonly in TEA as a cohesive set of difficulties, probably reflecting the involvement of relevant memory systems in this condition.

However, TEA remains a controversial disorder. Through our project website (The Impairment of Memory in Epilepsy (TIME Project) http://projects.exeter.ac.uk/time/, we receive contacts from patients around the world who have self-diagnosed, often following initial misdiagnosis, or have found it difficult to locate a clinician familiar with the disorder. Initial misdiagnoses, in patients later shown to have TEA, have included TGA, psychogenic amnesia, transient ischaemic amnesia, incipient dementia and sleep inertia. The TEA-associated interictal memory deficits are also under-recognized: clinicians continue to reassure concerned patients that their memory is normal in the absence of tests of long-term retention or remote memory, which can reveal otherwise undetectable but relevant memory impairments.^9^

This paper has two primary aims. First, to consolidate the scientific description of TEA, we present a new series of 65 patients with TEA [The impairment of memory in epilepsy study: Phase 2 (‘TIME2’) case series] with whom to compare the core clinical features identified from our previous series of 50 patients [the impairment of memory in epilepsy study: Phase 1 (‘TIME1’) case series].^5^ In doing so, we seek to confirm previous observations regarding demographic, seizure characteristics and interictal features and then combine the cohorts to provide an updated set of data based on this larger series of 115 patients. To determine whether these core clinical features are then observed in other studies of TEA, we also review relevant publications from other research groups, post-dating our earlier review of the topic.^4^

Second, following the consolidation of all current evidence regarding the description of TEA, we propose a novel disease model, with discussion around key uncertainties about TEA, and important questions for future research.

## Materials and methods

### Participants

#### Patients

Cases of TEA were recruited to the TIME (The Impairment of Memory in Epilepsy) study, using Zeman et al.^7^ diagnostic criteria:

1. A history of recurrent witnessed episodes of transient amnesia;
2. Cognitive functions other than memory are intact during typical episodes as observed by a reliable witness; and
3. Other evidence for a diagnosis of epilepsy. This can be provided by any combination of:
  - Epileptiform abnormalities in electroencephalogram (EEG),
  - The concurrent onset of other clinical features of an epileptic seizure (e.g., lip-smacking and olfactory hallucinations), or and
  - A clear-cut response to antiepileptic drugs.

Patients in TIME2 were either referred to the study via a consultant neurologist (*n* = 53), or self-referred (*n* = 12) after reviewing our project website (https://projects.exeter.ac.uk/time/). In this study, we include only participants referred to our study who were available for clinical assessment and—in most cases—neuropsychological assessment in the UK.

The study was approved by the Multicentre Research Ethics Committee, United Kingdom (MREC 03/10/77). All patients gave written, informed consent in accordance with the Declaration of Helsinki.

#### Control participants

We draw on two sets of control data. For standard neuropsychological measures, mood measures and for the assessment of autobiographical memory, data previously collected from the 24 healthy controls recruited in the TIME1 series were used again for comparison in TIME2. For measures of accelerated long-term forgetting, where some adjustment in administration procedures occurred between TIME1 and TIME2 (see Neuropsychological Assessment below), a new cohort of controls was recruited. This involved 22 age and IQ-matched healthy adults from the Exeter and Oxford areas.

### Clinical interview

Interviews were conducted by a member of the study team. A detailed history was obtained from the patient and at least one witness. A standardized data-collection pro forma was used to collect information in relevant domains (demographics, clinical features of the amnestic attacks, interictal symptoms, past medical history, past psychiatric history, epilepsy risk factors, current medications, family history). Medical case notes and correspondence were reviewed.

### Clinical investigations

EEG and magnetic resonance imaging (MRI) reports were requested from the referring clinical teams.

### Neuropsychological assessment

#### Standard measures

Participants were invited to complete a comprehensive neuropsychological assessment. This involved the same test battery as our original study, comprising measures of: general intelligence (Wechsler Abbreviated Scale of Intelligence-2 (WASI) subtest version^10^), anterograde memory (immediate and 30-min delayed recall of prose passage 1 from Wechsler Memory Scale-III^11^; copy and 30-min delayed recall of the Rey–Osterrieth complex figure (RCFT)^12^; the recognition memory test (RMT)^13^), language (graded naming test)^14^ and executive function (letter and category fluency).

#### Accelerated long-term forgetting

To take account of methodological recommendations made elsewhere^15^ we modified the method used to assess accelerated forgetting in our previous study.^5^ In this second cohort, the threshold for learning was lowered from 90% to 80%, with no minimum number of trials (to remove overlearning), and a verbal ‘wash-out task’ was included to reduce the impact of verbal working memory on performance.

A list of 15 words (from the Rey Auditory Verbal Learning Task) was presented orally over a maximum of 10 trials until at least 12 words (80% accuracy) could be recalled within a given learning trial. Upon reaching this criterion, participants were instructed to count backwards out aloud from 100 for 40 s, to prevent rehearsal of words. Recall of the words was assessed immediately following this distractor task, and at delays of 30 min and at 1 week (via telephone). After this last free recall trial, recognition memory was tested using the standard list of 30 words read aloud by the examiner (wherein the 15 targets are intermixed with 15 foils). The participant is asked to indicate for each word whether it had been in the original list or not. Scores were determined by the correct identification of words on the original list (leading to a maximum score of 15). Although participants were not forewarned about the delayed probes, participants were asked not to practice or write down the words between the face-to-face testing session and the telephone follow-up.

#### Remote memory

Autobiographical memory was assessed using the modified autobiographical memory interview (MAMI), as in our previous study.^5^ This semi-structured interview requires participants to describe two events, relating to specific topics (e.g. holidays, weddings, career changes, car ownership and hobbies) from each decade of their lives (from their 20s through to their current decade). For each event described, participants answer 5 questions designed to test their personal semantic memory (e.g. what type of car did you own in your twenties?) and then produce one detailed episodic memory (e.g. can you recall one time when you broke down or took it to get repaired/serviced?). Each episodic memory is scored out of 5, based on the scheme described previously^16^ where a score of 0 indicates a failure to recall a relevant memory and 5 indicates successful retrieval of a specific episode in which event details are described. This generates a personal semantic score (out of 10/decade) and an episodic score (out of 10/decade).

### Mood

Self-reported symptoms of depression or anxiety were measured through the hospital anxiety and depression scale.^17^

### Statistical analysis

Statistical analysis of data obtained through neuropsychological testing was performed using IBM SPSS Statistics 25.0. Analyses of variance (ANOVA) were conducted to compare groups’ performances (TIME1, TIME2 and controls). Planned contrasts comparing: (i) TIME2 participants with controls and (ii) TIME1 participants with TIME2 participants were included. We applied a Bonferroni adjustment of alpha (0.05/11) = 0.0045 to correct for the number of neuropsychological measures compared in each instance.

To examine any change in frequency of detected memory impairments across the two patient cohorts, a Pearson’s chi-squared test was used to compare the number of cases identified in each patient cohort (where cases of memory impairment were defined by performance 2 or more standard deviations (SDs) below the control mean on more than one neuropsychological test).

To investigate long-term anterograde memory performance, word list recall scores were compared between TIME2 and a new, matched control group (see above) via a repeated-measures ANOVA, with factors of participant group (TIME2 or control) and delay interval (40 s, 30 min and 1 week). The Huynh–Feldt correction for nonsphericity was applied, where needed. Given the differences in procedure, no direct comparisons were made between the TIME1 and TIME2 data sets.

Participants were entered into this analysis if they were not impaired on standard measures of memory, had satisfied the learning criterion (80% recalled) and demonstrated adequate retention over 30 min (recalling 8 or more words, consistent with a performance >1.5 SD from the mean in a normative study^18^) This was to ensure that accelerated long-term forgetting was not over-estimated within the sample due to poorer initial encoding and consolidation over shorter time periods.

To evaluate autobiographical memory in our second cohort, the semantic and episodic memory scores per decade from the MAMI were analysed using repeated-measures ANOVA, with a between group factor of participant group (TIME2 or control) and a within group factor of decade (20s, 30s, 40s, 50s and most recent). For the one participant whose current decade and epilepsy onset were both in the 50s, data were not included in the analysis of the ‘50s’ decade, but appeared under ‘most recent’ decade, to reflect acquired after the onset of TEA. To examine autobiographical memory performance for events that occurred prior to the acute disturbances in memory due to epilepsy onset, only patients with an age of TEA onset >50 years were entered into this analysis. A separate comparison of recent memory (from each individual’s current decade) was also conducted.

Lastly, self-reported symptoms of anxiety and depression were analysed using ANOVA to compare TIME1, TIME2 and healthy controls.

### Literature search

We performed a literature search using the following keywords: ‘transient epileptic amnesia’ in MEDLINE, Embase and PsycINFO up to September 2018. Studies published prior to 2008 were excluded as these had been analysed in a previous review article.^4^ Titles and abstracts were reviewed and further hand-searching using reference lists was performed to identify additional published papers. Conference abstracts were not included in the analysis, nor were articles published in a language other than English.

The data that support the findings of this study are not publicly available due to privacy and ethical restrictions.

## Results

### Clinical features in TIME2 patients

#### Demographics

A total of 65 patients (51 males, 14 females) were recruited between January 2008 and April 2016. Mean age at the onset of amnestic attacks was 61.4 years (SD 9.95; range, 26–77 years), and at entry into the study was 65.6 years (SD 8.67; range, 39–81 years).

#### Diagnostic criteria

**Figure 1:**
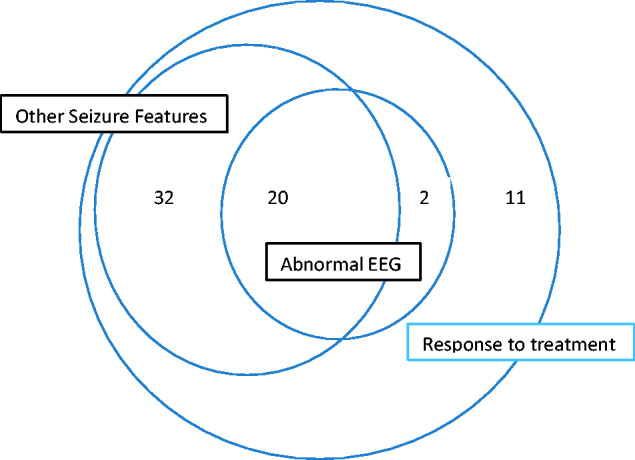
Frequency of cases meeting criteria for diagnosis of TEA in TIME2 series patients.

The grounds for the diagnosis of TEA in each case are summarized in Fig. 1. Case-by-case details of diagnostic criteria are provided in Supplementary Table 1.

TEA was the initial diagnosis in only 40% of cases (26/65). Other initial diagnoses were: temporal lobe epilepsy (18/65), TGA (10/65), transient cerebral ischemia/stroke (6/65) and ‘psychogenic’ (5/65). The median delay between first amnestic episode and diagnosis of TEA was 4 years (mean, 4.17; interquartile range, 3–5).

#### Seizure features

##### Duration, timing and frequency

Median attack duration was 15–30 min, with a wide variation (range <1 min to days—see Supplementary Fig. 1). A total of 61/65 patients (94%) reported that at least some of their attacks occurred on waking from sleep. However, only 5 patients exclusively experienced seizures at this time. Median frequency of attacks per annum prior to diagnosis was 12 (interquartile range 8–20). Median total number of attacks experienced prior to diagnosis was 15 (interquartile range 6–36).

##### Seizure types

Amnesia was the sole ictal manifestation in 13/65 (20%) of patients. In 33/65 (51%) brief unresponsiveness was reported in at least some attacks. 14/65 (22%) reported an epigastric aura. Olfactory hallucinations were described in 29/65 (45%); the same percentage reported motor automatisms, most commonly repetitive chewing or swallowing movements (11/65, 17%). Tonic–clonic seizures occurred in only 7/65 (11%) and were typically isolated or rare events.

##### Ictal amnesia

A total of 30/65 (46%) patients had dense ictal amnesia while the remaining 35/65 (54%) patients were able, on some occasions at least, to ‘remember not being able to remember’—i.e. had partial recall of their transient amnestic episodes. Repetitive questioning during episodes occurred in 41/65 (63%) cases.

##### Treatment

All patients were started on anticonvulsant medication, with 92% reporting complete cessation of attacks. The most commonly used final medications were lamotrigine (31/65; 48%) followed by levetiracetam (14/65; 22%), carbamazepine and sodium valproate (10/65 or 15% each). Topiramate was used in 3 patients (3/65, 4.6%) and zonisamide was used in one patient (1/65, 1.5%). Drug changes were required in 24 patients (37%), either due to inefficacy or side effects of the initial medication. This included changes from carbamazepine in 12 patients, lamotrigine in 9 patients, sodium valproate in 8 patients, levetiracetam in 4 patients and phenytoin in 2 patients. Final median daily doses were: lamotrigine 150 mg (range 50–300 mg), levetiracetam 1000 mg (500–3000 mg), carbamazepine 500 mg (300–1600 mg), sodium valproate 900 mg (400–2400 mg), topiramate 100 mg (50–150 mg) and zonisamide 400 mg (400 mg). At the time of interview, 59/65 patients (90.8%) were on antiepileptic monotherapy, the remainder taking two medications.

#### Interictal features

##### AbA

A total of 57 patients (88%) reported AbA, ranging from patchy losses for the previous 1–2 years to loss of memories up to 30 years in to the past. These forgotten episodes were frequently noted in conversation with friends and family and typically included shared experiences such as weddings, holidays and birthdays.

##### ALF

A total of 48 patients (74%) reported ALF, either without prompting or when asked if they had experienced memories fading more quickly that they would typically expect over hours to weeks.

##### TopA

A total of 47 patients (72%) reported TopA.

##### Olfaction

A total of 29 patients (45%) reported ictal olfactory hallucinations. A total of 16 (25%) reported a reduction in their sense of smell. Overall 34 patients (52%) reported olfactory symptoms of some kind.

##### Emotional lability

A total of 26 patients (40%) reported a state of emotional lability, principally involving a tendency for sadness/tearfulness to be provoked by relatively minor stimuli (24/26), and sometimes also a feeling of increased irritability (4/26).

#### Investigations

##### MRI

Fifty-eight participants underwent a clinical MRI scan. Clear abnormalities were detected in 4 patients: (i) high T_2_ signal in the right hippocampus; (ii) frontal encephalomalacia secondary to previous brain injury, (iii) slight T_2_ signal change in both hippocampi and (iv) small cystic lesion in the right caudate with small area of gliosis right lateral ventricle and left posterior frontal lobe. A further two patients were noted to have evidence of vascular disease (mild microvascular ischaemia, past minor vascular event).

##### EEG

Sixty-one patients had undergone interictal EEG. Overall, 22/61 (36%) were epileptiform, 17/61 (28%) showed borderline abnormalities and 22/61 (36%) were normal. Epileptiform discharges localized to the temporal lobes, primarily, or solely, in the left hemisphere in 13/22, right-sided in 5/22 and bilateral in 4/22. Non-specific abnormalities most often involved theta activity, usually localized to the temporal lobes (11/17). Other non-specific abnormalities included a single sharp wave (*n* = 5) or a single sharp-slow complex (*n* = 2). These borderline abnormal findings were focused bilaterally (7/17), on the right (7/17) or on the left (3/17) (Supplementary Fig. 2).

#### Comparison with clinical features in TIME1 series

Table 1 presents the clinical features in the current series, our previous series and the two series combined.

**Table 1:** Comparison of key features in TIME1 and TIME2

Core clinical features of TEA	TEA 2007 (*n* = 50)	TEA 2017 (*n* = 65)	*P*-value	TEA combined (*n* = 115)
Demographics				
Mean age at onset (years)	62.1 (range 44–77) (SD 9.1)	61.4 (26–77) (SD 9.95)	0.872	61.7 (26–77)
Mean age at presentation (SD)	66 (SD 9)	65.6 (SD 8.67)	0.150	66.7
Sex distribution (M/F)	34/16	51/14	0.207	85/30
Seizure characteristics				
Median number of attacks prior to diagnosis	10 (IQR 6–30)	15 (IQR 6–36)	0.263	12 (IQR 6–25)
Median frequency of attacks (per year)	12 (IQR 5–20)	12 (IQR 8–20)	0.953	12 (IQR 5–12)
Median attack duration	30–60 min (range <1 min to days)	15–30 min (range <1 min to days)		15–30 min (range <1 min to days)
Cessation of attacks on AED (%)	96	91	0.294	93
Amnesia sole manifestation of a seizure (%)	28	20	0.318	24
Tonic–clonic seizures (%)	4	11	0.186	8
Some attacks on waking (%)	74	94	0.003^*^	85
Partial amnesia for attack (%)	56	54	0.840	55
Repetitive questioning (%)	50	63	0.164	57
Olfactory hallucinations (%)	42	45	0.749	43
Motor automatisms (%)	36	45	0.333	41
Brief unresponsiveness (%)	24	50	0.005^*^	39
Interictal features (%)				
c/o autobiographical memory loss	70	88	0.017^*^	80
c/o accelerated forgetting	44	74	0.001^*^	61
c/o topographical memory loss	36	72	<0.001^*^	56
Emotionality	18	40	0.011^*^	30
Investigations (%)				
Interictal epileptiform activity on EEG	37	33	0.656	31
Structural lesion on MRI	2	7	0.124	5

AED = antiepileptic drug; AML = autobiographical memory loss; c/o = complains of; EEG = electroencephalogram; F = female; IQR = interquartile range; M = male; MRI = magnetic resonance imaging.

* A significant difference between groups (*P* < 0.05). Cohorts compared using Pearson’s chi-square testing.

#### Demographics

Age at seizure onset and sex ratio were consistent between the two series, resulting in a mean onset age of 62 years, and a male predominance (overall 74% men).

#### Seizure features

##### Duration, timing, frequency

Seizure duration and frequency were similar in the two series, with 12 brief attacks typically reported per year. Seizures on waking were reported more often in TIME2, with an increase from 74% to 94% of cases (*P* = 0.003).

##### Seizure types

The frequency of repetitive questioning, olfactory hallucinations and motor automatisms were similar across the two patient groups (with overall frequency ranging between 41% and 57% of patients). Pure amnestic seizures and tonic–clonic seizures also occurred with similar frequency in the two series, in 24% and 8% of patients respectively. There was an increase in reports of brief episodes of unresponsiveness across the two cohorts, half the cases reporting this in TIME2 compared to a quarter of cases in TIME1.

##### Ictal amnesia

Partial recollection of attacks occurred in around half of the patients in both groups.

##### Treatment

Over 90% of patients in both series reported complete cessation of seizures following the initiation of medication.

#### Interictal features

##### AbA, ALF, TopA

These specific interictal memory disturbances were reported more often in TIME2 (all *P* < 0.05), with almost twice as many patients reporting accelerated forgetting (74%) and topographical amnesia (72% of cases) as in TIME1.

##### Emotional lability

Increased emotionality was also reported more often in TIME2 than it had been in TIME1 (*P* = 0.011).

#### Investigations

EEG and MRI abnormalities were seen with similar frequency in the two series, with clear epileptiform activity observed in approximately a third of cases, but structural lesions occurring rarely (5% of patients overall).

### Neuropsychology

#### Standard neuropsychology

Neuropsychological test results for patients in TIME1, TIME2 and control participants are shown in Table 2. While full assessments were conducted in 56 participants, three were excluded from analysis given other neurological history which may have confounded test performance (one because of significant head injury resulting in structural changes evident on MRI; and two because of evidence of past vascular events evident on MRI).

**Table 2:** Neuropsychological test performance (mean and standard deviation)

Neuropsychological measure	TIME2 (*n* = 53)	TIME1 (*n* = 50)	Controls (*n* = 24)
Weschler abbreviated scale of intelligence (WASI) (2-subtest IQ)	115.7 (14.8)	118.3 (12.8)	120.0 (14.4)
Graded naming test (/30)	21.5 (4.8)	21.4 (5.1)	23.5 (4.2)
Controlled oral word association test (COWAT) (Letters F, A, S)	41.8 (13.6)	42.5 (13.9)	43.8 (11.4)
Animal fluency	19.5 (6.9)	19.3 (5.9)	22.0 (4.4)
Rey-Osterrieth complex figure tes (RCFT)—copy (/36)	33.5 (3.5)^*^^,a^	34.5 (3.1)	35.5 (1.1)
Logical memory (LM) (Story 1)—immediate (/25)	11.5 (4.1)^**^^,b^	14.0 (4.3)	15.9 (3.8)
LM (story 1)—delay (/25)	9.1 (4.7)^**^	11.7 (5.0)^*^	14.7 (3.8)
LM (Story 1)—recognition (/15)	12.1 (2.0)^**^	12.9 (1.4)^*^	13.6 (1.2)
RCFT—30 min delay (/36)	15.3 (5.9)^**^^,^^a^	15.0 (6.5)^*^	18.6 (6.1)
Recognition memory test (RMT)—words (/50)	43.3 (6.1)^**^	46.1 (4.7)^*^	48.3 (1.9)
RMT—faces (/50)	39.4 (5.3)^**^	40.7 (5.4)^**^	45.1 (2.9)

Healthy control participants from Butler et al.^5^ ANOVA conducted to compare groups’ performances. A Bonferroni adjustment of alpha (0.05/11) = 0.0045 to correct for the number of neuropsychological measures compared in each instance

a Based on a sample of *n* = 50.

b A significant difference *P* < 0.004 between TEA cohorts across TIME1 and TIME2.

* A significant difference *P* < 0.05 when compared with healthy controls.

** *P* < 0.0045.

All three groups of participants demonstrated above average intellectual ability. There were no significant differences between TIME2 and healthy controls on language or executive function tasks, however, significant reductions were apparent on all of the anterograde memory tasks (*P* < 0.0045).

#### Accelerated long-term forgetting

ALF testing was completed per protocol in 36 of the TIME2 participants (with an additional 15 participants having either been administered a different version, or having not completed the full protocol, and 2 participants having not been administered the task). Thirteen were excluded from analysis due to impaired performance on standard tests of anterograde memory (delayed story recall). A further 4 failed to meet the word list learning criterion, and an additional patient performed poorly at the 30-min interval. Nineteen TEA participants and 22 age and IQ-matched healthy controls were therefore included, subject to the same exclusions listed above (healthy control mean age = 63.82, TIME2 subset mean age = 64.77 *F*[1, 41] = 0.30, *P* = 0.59; healthy control mean IQ = 116.86, TIME2 subset mean IQ = 116.38, *F*[1, 41] = 0.87, *P* = 0.36). All participants completed between 3 and 10 learning trials, with no differences between the TEA and control groups on the total number of learning trials (*F*[1, 40] = 0.58, *P* = 0.45), or average final trial score (85%; *F*[1, 40] = 0.01, *P* = 0.93), suggesting an equivalent performance during the learning phase of the task.

**Figure 2:**
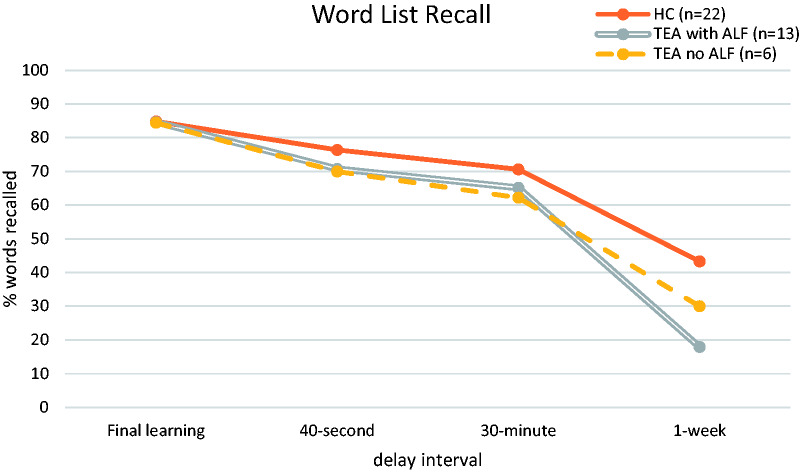
**Mean word list recall performance of TEA patients and matched control participants over testing intervals (TIME2).** Repeated-measures ANOVA of recall performance confirmed a significant main effect for group (*F*(1,39) = 10.46, *P* = 0.002), a significant main effect for the delay interval (*F*(1.5,58.9) = 153.73, *P* < 0.001), and, importantly, a significant group × delay interaction (*F*(1.5,58.9) = 6.29, *P* = 0.008). Planned contrasts to explore group differences across the time intervals showed that, while the TEA and control participants did not differ significantly from each other in their change shown between 40-s and 30-min recall (*F*(1,39) = 0.03, *P* = 0.86), the degree of forgetting was greater in TEA participants compared to controls when comparing the words recalled at the short delay intervals (i.e. averaged across 40-s and 30-min recall intervals) and performance at the 7-day interval (*F*(1,39) = 7.74, *P* = 0.008).

As expected, recall performance declined over time for both TEA and control participants (see Fig. 2 for mean group results), with the lowest scores generated by the TEA group at all delay intervals. Repeated-measures ANOVA of recall performance confirmed a significant main effect for group (*F*(1, 39] = 10.46, *P* = 0.002), a significant main effect for the delay interval (*F*(1.5,58.9) = 153.73, *P* < 0.001), and, importantly, a significant group × delay interaction (*F*(1.5,58.9) = 6.29, *P* = 0.008). Planned contrasts to explore group differences across the time intervals, showed that, while the TEA and control participants did not differ significantly from each other in their change shown between 40-s and 30-min recall (*F*(1,39) = 0.03, *P* = 0.86), the degree of forgetting was greater in TEA participants compared to controls when comparing the words recalled at the short delay intervals (i.e. averaged across 40-s and 30-min recall intervals) and performance at the 7-day interval (*F*(1,39) = 7.74, *P* = 0.008) (see Fig. 2).

Within the TEA group, 13 participants had reported symptoms of ALF. To explore whether the group × delay interaction was only evident within these participants, additional separate analyses were run comparing control performance firstly with TEA participants who did (ALF+) and did not (ALF−) self-report ALF. As predicted, the interaction remained significant for ALF+ patients versus healthy controls [*F*(1.53,66) = 8.12, *P* = 0.002, with contrasts confirming that this effect was only significant at the final level of delay (*P* = 0.003) and not at early intervals (*P* = 0.97)] but was no longer significant when comparing ALF− patients with healthy controls (*F*(1.57,40.92) = 0.40, *P* = 0.533, with a significant main effect for delay, but no main effect for group).

Although it was not possible to compare recognition memory scores of TEA and control participants, as control data were not collected, 8 of the 19 patients with TEA performed at or below a score of 9, a level sometimes used to differentiate performance above chance. An additional participant refused the task after being unable to recall any words on free recall. Scores ranged from 7 to 14 out of 15 (mean = 10.33, SD = 2.38), with only 3 TEA participants scoring above 12.

Finally, to check for any associations among seizure variables (total number of seizures and frequency of seizures prior to anti-convulsants) and long-term retention, Spearman’s rho correlations were examined. There were no significant results (1-week retention and total seizures prior to anti-convulsants: rho = −0.11, *P* = 0.662; 1-week retention and seizure frequency prior to anti-convulsants: rho = −0.10, *P* = 0.749). We note, however, that patients were seizure free at the time of testing.

#### Autobiographical memory

The MAMI was conducted with 24 TEA participants (17 M, 7 F) from TIME2 who met the criterion of TEA onset from age 50 years onwards. As in TIME1, eligibility also required participants to show adequate performance (within 2 SDs of the mean of the healthy control group) on standard anterograde memory tests (i.e. delayed story recall). Individuals included did not differ on demographic (current age, sex, years education), clinical (years since onset, time to diagnosis, yearly frequency of attacks) or non-memory neuropsychological measures (IQ, naming, verbal fluency, visuospatial) from those in TIME2 who were not tested on the MAMI. Those included reported slightly fewer symptoms of depression on the hospital anxiety and depression scale, but this did not signify a clinically meaningful difference as both groups were within the normal limits. TEA participants were age and IQ-matched with 18 healthy controls from TIME1 (TEA mean age = 67.83, control mean age = 68.17, *P* = 0.881; TEA mean IQ = 120.08, control mean IQ = 121.50, *P* = 0.735).

**Figure 3:**
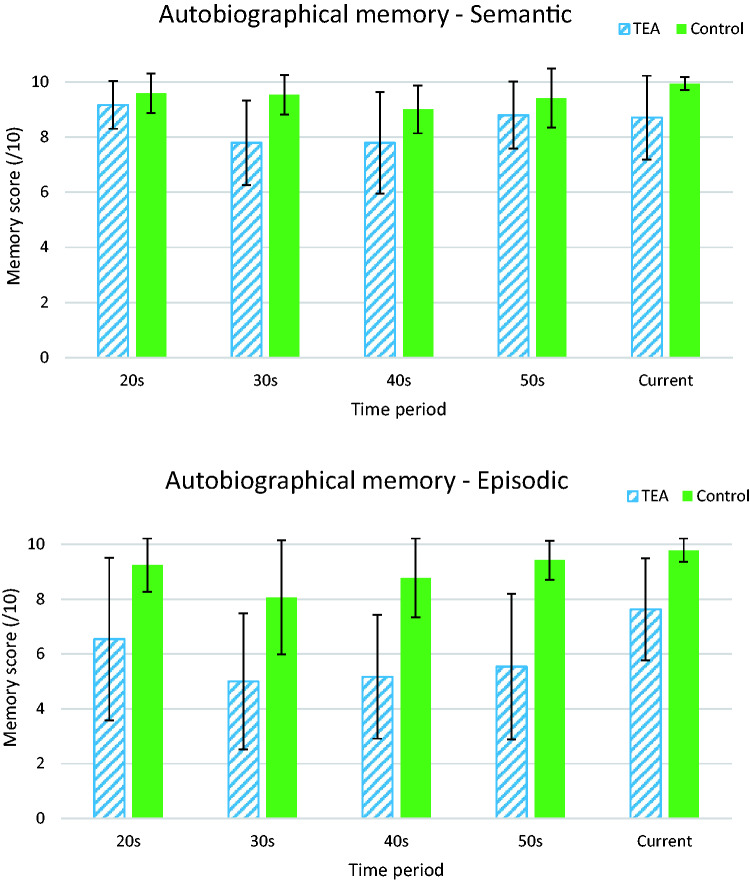
**Mean scores on the MAMI for TEA patients and matched control participants over decades.** Error bars are SDs. Repeated-measures ANOVA revealed a significant main effect for decade (*F*(2.68,104.61) = 5.758, *P* = 0.002). This followed a quadratic function (*F*(1,39) = 10.92, *P* = 0.002) such that memories for the 20s and 50s were better recalled than for the middle decades. A significant main effect was also found for group (*F*(1,39) = 20.98, *P* < 0.001), with average personal semantic recall for controls slightly higher, at 9.58 out of 10, as compared with 8.53 out of 10 for TEA participants. However, no decade × group interaction (*F*(2.68,104.61) = 2.65, *P* = 0.059) was observed, indicating that the pattern of performance of TEA participants, while lower, mirrored that of the controls. In the episodic domain, a significant main effect arose for decade (*F*(3,117) = 4.39, *P* = 0.006), again with contrast testing confirming a quadratic relationship where memories from the 20s and the 50s were better recalled that those from the middle periods (*F*(1,39) = 11.95, *P* = 0.001). The main effect for group was also significant (*F*(1,39) = 45.20, *P* < 0.001), with average episodic recall remaining high for controls at 9.14 out of 10, but dropping to an average score of 6.23 out of 10 for TEA participants. The decade × group effect was not significant (*F*(3,117) = 0.87, *P* = 0.457), indicating that the overall pattern of performance across the decades was similar for the two groups.

Figure 3 shows the mean scores by decade for the two groups. For the personal semantic memory component of the test, repeated-measures ANOVA revealed a significant main effect for decade (*F*(2.68,104.61) = 5.758, *P* = 0.002). This followed a quadratic function (*F*(1,39) = 10.92, *P* = 0.002) such that memories for 20s and 50s were better recalled than for the middle decades. A significant main effect was also found for group (*F*(1,39) = 20.98, *P* < 0.001), with average personal semantic recall for controls slightly higher, at 9.58 out of 10, as compared with 8.53 out of 10 for TEA participants. However, no decade x group interaction (*F*(2.68,104.61) = 2.65, *P* = 0.059) was observed, indicating that the pattern of performance of TEA participants, while lower, mirrored that of the controls. We note that the high mean score of controls on our measure of personal semantic memory raises the possibility of a ceiling effect, which could lead to some underestimation of the corresponding deficit among patients

Similar but more striking results were found in the episodic domain. A significant main effect arose for decade (*F*(3,117) = 4.39, *P* = 0.006), again with contrast testing confirming a quadratic relationship where memories from the 20s and the 50s were better recalled that those from the middle periods (*F*(1,39) = 11.95, *P* = 0.001). The main effect for group was also significant (*F*(1,39) = 45.20, *P* < 0.001), with average episodic recall remaining high for controls at 9.14 out of 10, but dropping to an average score of 6.23 out of 10 for TEA participants. The decade x group effect was not significant (*F*(3,117) = 0.87, *P* = 0.457), indicating that the overall pattern of performance across the decades was similar for the two groups.

To determine the proportion of TEA participants who at an individual level showed impaired performances at each decade, cut-off scores were calculated using the threshold of 2 SDs below the control mean. Proportions varied from 38% of patients (for the 30s decade) up to 79% (for the 50s decade), with only two participants (13%) not classified as impaired on any of the examined decades. Thus, impairments were highly prevalent and observed across the lifespan.

Finally, to check for any associations among seizure variables (total number of seizures and frequency of seizures prior to anti-convulsants) with overall autobiographical memory performance, Spearman’s rho correlations were examined. There was no significant relationship between average episodic memory performance on the MAMI and frequency of seizures prior to anti-convulsant medication (rho = −0.11, *P* = 0.624), but there was a significant, negative association between average episodic MAMI score and total number of attacks (rho = −0.41, *P* = 0.047), indicating that those who had experienced more seizures showed poorer autobiographical memory.

#### Mood

Participants reported relatively few symptoms of anxiety or depression (see Table 2). While no statistical differences were observed when comparing mean depression scores from healthy controls with TIME2 participants (*t* (68.78) = −1.538, *P* = 0.129) there was a statistical difference regarding reported symptoms of anxiety (*t* (65.338) = −2.219, *P* = 0.03). This numerical difference, however, is not clinically significant as both groups reported mean levels below the standard clinical cut-offs (<8).

#### Comparison with neuropsychological and mood features in TIME1 series

Performance of TIME1 and TIME2 cohorts was similar (all *P* > 0.16) on standard neuropsychological tests except that the TIME2 group performed more poorly on the immediate recall of a short story (*t* (101) = −3.05, *P* = 0.003). At the individual level, a third of TIME2 participants (34%) showed significant memory impairment (>2 SDs below the control mean on 2 or more memory tests). Although a slightly higher number than observed in the TIME1 cohort (28%), the difference was not significant (*n* = 103; χ^2^ = 0.427, *P* = 0.531). No differences were observed in self-reported symptoms of anxiety (*P* = 0.159) and depression (*P* = 0.216). Thus the findings of standard neuropsychological tests and mood of TIME1 appeared largely replicated in this second cohort.

The findings with respect to ALF and AbA are also broadly similar in the two groups. Subtle differences in test administration and patient selection mean that the results are not exactly comparable, but there was clear evidence in both study groups of (i) an anterograde memory impairment that—in some cases—became apparent only at longer than standard delays and (ii) an autobiographical memory impairment affecting memory for both semantic and episodic details, the latter more severely, both occurring in participants with normal memory performance on standard tests.

#### Literature review

Using the search term ‘Transient Epileptic Amnesia’, we identified 322 publications between 2008 and 2018, after deduplication (Supplementary Fig. 3). The results were filtered to include only case series and case reports of novel cases of TEA: this yielded in total, 102 patients with TEA from 23 studies, which are summarized in Table 3, excluding the cases from our previous and current studies. All cases satisfied the Zeman et al.^7^ criteria for TEA.

**Table 3:** Literature review of TEA case reports and series since 2008

Author	Year	*N*	M	F	Mean age (onset)	Mean age (diagnosis)	Duration of attacks	Attacks on waking	Ictal AA	Olf hall	Automatisms	Unresp	EEG (epileptiform changes)	Imaging	Response to AED
Huang	2008	1	1	0	67	67	5 h						L	Poss metastatic tumour L TL	
Hornberger	2010	1	0	1	43	44	<1 min			No	No	No	R	MRI normal	Complete
Razavi	2010	1	1	0	67		A few minutes						B	MRI normal	Complete
Ioannidis	2011	3	1	2	53, 62, 73	54, 65, 75	30–45 min	Yes (in 2)	Yes	Yes ×1	No	No	R	R TL angioma, R ant choroidal aneurysm, MRI normal	Complete
Favre	2011	1	0	1	60	70	30–60		Yes				R	MRI normal	Complete
Soper	2011	1	0	1	45	47	1–15 min		Yes		Manual	No	R	Asymm HC	Complete
Walsh	2011	1	1	0	55	59		Yes		No	No	Yes	L	MRI normal	No
Kemp	2012	1	1	0	20-year history	73	1–10 min	Yes	Yes		Oral			MRI normal	Complete
Mosbah	2014	30	18	12	59 (43–77)		<5 min to >1 h	In 23%	In 20%	2/30	Oral in 4/30	2/30	17/30 ep.	Normal in 70% (R parietal lobe (PL) meningioma, R hemisphere ischaemic sequelae, atrophy (cortical, R hippocampal), hyperintensity (bilat hippocampal, R amygdala)	Complete in 19 (73%) of 26 cases, >50% reduction in seizure frequency in the remaining cases (27%).
Lapenta	2014	11	7	4	54.9 (35 to 78)	59.7yrs	2–10 min		Yes	1/11	Oral in 1/11	3/11	7/11 ep.	MRI normal in 3, 5 MTL signal abnormalities	Complete in 10/11
Del Felice	2014	3	2	1		71.25 (67 to 75)	1 min to 1 h		Yes (in 2)	Yes	Oral	1/3	N	Subtle mesial temporal lobe (MTL) atrophy	Complete
Nicastro	2014	1	0	1	79	79	90 min	Yes	Yes		No	No	B	MRI normal	Complete
Cretin	2014	1	0	1		64	20–60 min		Yes		Oral + manual	Yes	N	CT and MRI normal	Complete
Woollacott	2015	1	0	1	74	76	1 h	Yes	Yes		Oral	Yes	L	CT—temporal lope (TL) atrophy	Levetiracetam—no, Lamotrigine—complete
Sugiyama	2015	1	0	1	75	5 months later	30 min	Yes	Yes	No	No	Yes	B	MRI—small hyperintense lesion in R TL	Complete
Cunha	2016	3	0	3		74, 67, 70							L	MRI normal	
Fouchard	2016	1	0	1	63	68	1–2 h	Yes	Yes		No	No	L	MRI—enlarged hippocampal volume	Complete
Cho	2017	2	1	1		77, 63	10–20 min	Yes	Partial		No	No	R	MRI normal	Complete
Burkholder	2017	2	0	2	12–18 months	50, 59	2–8 h	Yes	Yes			½	B	MRI normal	Complete
Sekimoto	2017	1	0	1	6/12 earlier	67	15 min	Yes	Yes	No	No	No	R	MRI normal	Complete
Ukai	2017	1	0	1		67		Yes			No	Yes	B	MRI normal	Complete
Lanzone	2018	15	4	11	67.2 (59–75)		20 min to 24 h	In 40%	Yes				IEA in 15/15 on 24 hr EEG	MRI normal in 11/15 (thalamic cavernoma, cystic pinealoma, bifrontal post-traumatic lesion, L TL venous ectasia)	complete in 8/15, partial in 5/15
Ramanan	2019	19	14	5	66.6 (43–85		Minutes to several hours						12/19 ep.	MRI normal in 15/19 (small encephalocoele, foci of restricted diffusion L hippocampus, hemosiderin L temporal, splenium corpus callosum T_2_ hyperintensity)	Successful treatment in 18/19

### Clinical features

#### Demographics

Summing across these studies, the sex ratio was equal (males 51, females 51), though three of the four larger series reported a male preponderance.^6^^,^^19^^,^^20^ The mean age of onset for TEA has been reported as 59, 67.2 and 66.6 years in the three larger series providing this information, with onset age ranging from 35 to 85 years. The reported interval between symptom onset and diagnosis ranges from around 6 months to 6.2 years.

#### Diagnostic criteria

##### Seizure features

###### Duration

The majority of reported episodes fall between a few minutes and 1 h, but there is extensive variation with seizure episodes lasting from <1 min^21^ up to 24 h.^22^

###### Timing

A total of 13/23 (57%) studies included in this review describe episodes of TEA occurring on waking in 27/60 (45%) patients.

###### Frequency

The frequency of TEA attacks ranges from several times per week^23^ to less than once per year.^24^ Comparable data are available for 15/23 studies listed above (46/114 cases). In this group, 6/46 (13%) report at least one seizure per week, 17/46 (37%) report seizures at least once per month but less than weekly and 23/46 (50%) describe less than one seizure per month.

###### Seizure types

Pure amnestic seizures are described in between 17%^6^ and 64%^19^ of patients. Brief unresponsiveness is reported in a total of 9/23 studies. A total of 12/51 (23.5%) cases presented by these studies describe this phenomenon. An epigastric aura is described in three studies^25–27^ and orofacial automatisms in four.^24–27^ Generalized tonic–clonic seizures are uncommon [10% in Mosbah,^6^ 9% in Lapenta^19^]. In one case,^28^ persistent generalized tonic–clonic seizures were resistant to anti-epileptic treatment leading to a temporal lobectomy.

###### Treatment

Patients with TEA respond well to treatment with anti-epileptic medication. 94/96 cases (97.9%) in whom the response to anti-epileptic treatment was reported describe a reduction in the number of seizures following initiation of medication. In 59 of these patients, this is documented as being complete seizure cessation, and in 12, this reduction is described as partial (>50% reduction in seizures). Ramanan et al.^20^ states that 18/19 patients improved—although it is not clear whether this represents a partial or complete improvement.

##### Interictal features

The interictal features described in TEA have not been routinely assessed in either case studies or case series of TEA patients and are therefore not as thoroughly described. Findings in studies where these features have been investigated are described below.

###### AbA

A total of 14/23 studies included in this review describe interictal autobiographical memory impairments. Mosbah et al.^6^ report that retrograde memory loss is greater for the episodic than the semantic component of autobiographical memory. Recent memories were especially severely affected, with measurable improvement in autobiographical memory for events from the past five years following treatment.^6^

###### ALF

A total of 10/23 studies describe the presence of ALF in TEA, in 25 patients. In studies with multiple patients, this feature was described in 16/30 (53%),^6^ 4/19 (21%)^20^ 1/3 (33%)^26^^,^^29^ or 1/2 (50%),^24^ giving a total of 22/54 (40.1%).

###### TopA

TopA is described in only 2/23 studies (2 patients); described as either ‘a tendency to lose her way even in familiar locations’^30^ or simply as ‘topographical amnesia’.^26^ None of these studies measured topographical memory formally examined using neuropsychological tests.

###### Olfaction

A decreased sense of smell, occurring in the setting of TEA was described in only 1 study (1 patient).^31^ One of the three cases described by Ioannidis et al.^26^ features reports of ‘strange and bad smells’ as an element of seizure episodes.

###### Emotionality

Two of the 23 studies describe a clear change in the emotional character of their patients with TEA. In one case, this change was becoming angry and short-tempered,^31^ and in the other low mood and depression was reported.^25^

##### Investigations

###### EEG

The rate of EEG abnormalities reported in published case series has exceeded 50% (57%^6^ and 64%^19^). EEG abnormalities have also been common in TEA case reports, most often occurring in the right temporal or fronto-temporal leads,^21^^,^^23^^,^^26^^,^^32^ although abnormalities are also frequently found on the left^28^^,^^33^ and bilaterally.^34^^,^^35^

###### MRI

In the largest TEA case series, the majority of participants have normal MRI scans (15/19,^20^ 21/30,^6^ 11/15^22^). Of the 98 cases where MRI results were reported, 73.5% were normal. Where MRI abnormalities have been described, these have most commonly involved the temporal lobes (18/26), with findings including mesial temporal lobe signal abnormalities,^19^ right temporal cavernous angioma,^26^ a small hyperintense lesion in right hippocampus,^36^ restricted diffusion left hippocampus^20^ and enlarged hippocampal volume with loss of architecture and increased hippocampal tail signal.^33^ Extra-temporal abnormalities have included bifrontal post-traumatic change,^22^ right parietal lobe meningioma^6^ and a right anterior choroidal aneurysm.^26^

## Discussion

The substantial series of patients described here, combined with those in the literature review, support the existence of a treatment-responsive epilepsy syndrome characterized by amnestic seizures, often occurring at roughly monthly intervals, typically lasting for 15–30 min, frequently manifesting on waking, and with onset in middle age; a high frequency of interictal memory deficits, especially ALF and AbA, and—principally in our case series—both ictal and interictal olfactory disturbance with a tendency to emotional lability, specifically easily provoked tearfulness. We will discuss in turn (i) whether TEA should be regarded as an epilepsy syndrome, (ii) the neuropsychological and neurobiological bases of the prominent associated interictal memory disturbance and (iii) a model designed to capture current understanding of the condition and to identify key unanswered questions for future research.

### Is TEA an epilepsy syndrome?

There are compelling grounds for concluding that epilepsy is the underlying cause of the disorder described here: our diagnostic criteria require the presence of one or more indicative features, specifically the detection of epileptiform change on EEG, the occurrence of other clinical phenomena suggestive of epilepsy, such as paroxysmal alteration of awareness or olfactory hallucinations, and/or a clear-cut response to anticonvulsant drugs. While opportunities to record EEG during an amnestic episode are exceptional, such recordings indicate that transient amnesia can occur both as an ictal and as an immediately postictal manifestation.^37^

If the diagnosis of epilepsy is accepted in these cases, do they belong to a distinctive epilepsy ‘syndrome’? Epilepsy syndromes involve a ‘complex of signs and symptoms that define a unique epilepsy condition’; the complex should involve ‘more than just the seizure type’ but is distinct from an ‘epilepsy disease’, a condition with a ‘single, specific, well-defined aetiology’.^38^ TEA precisely satisfies this definition, given its distinctive demographic features, ictal characteristics and interictal manifestations. As its aetiology is varied, it is not an ‘epilepsy disease’. In this section, we will consider some potential objections to this view, in particular inconsistencies between the features reported in the existing literature, the existence of atypical cases and the ‘grey zone’ between TEA and other forms of temporal lobe or limbic epilepsy. The question of aetiology is considered further in section (iii).

While our two consecutive series of patients with TEA display marked commonalities, in demographic features and ictal characteristics, they differ with respect to the reported frequency of episodes on waking, interictal memory disturbance and emotional lability. In each case, the frequency of these features was higher in TIME2 than in TIME1. While we attempted to gather clinical data in a consistent fashion over time, we suspect that the apparent increase in the frequency of these features reflects increased vigilance, stimulated by our initial findings, rather than any true difference between the patient groups. However, whether or not this is the case, these modest quantitative differences between the two series do not undermine the key elements of the syndrome, outlined above.

The 115 patients described from our centre broadly resemble those reported from other centres in most respects, in particular, age of onset and seizure characteristics. The interictal neuropsychological features of TEA have been reported less frequently in other reports than in ours, but both AbA and ALF have been described repeatedly. Olfactory disturbance and emotional lability are much more common in our series than in other reports: Whether this reflects a true difference, or a difference in ascertainment, is unclear.

Some cases in our current series are atypical with respect to age, length of amnesic episode, treatment resistance; one additional case reported provocation of amnesic episodes by exertion. Two cases (305, 340) presented below the age of 30, more than three SDs below the mean age at presentation. Case 305 satisfied all three diagnostic criteria, with attacks of typical duration; case 340 satisfied one criterion (clear-cut treatment response) with longer than usual attacks (1–2 h); both had attacks on waking. Case 305 had experienced 26 episodes, case 340 five episodes. 4 patients (241, 383, 257 and 346) had ‘TGA-like’ episodes of amnesia lasting >2 h: Three of these patients satisfied two criteria each (treatment response and the occurrence of other suggestive features, olfactory hallucinations in two cases, unresponsiveness in one), while case 257 satisfied one criterion (treatment response). All four patients had experienced >10 episodes, and three of the four described episodes on awakening. All four were in their fifties. Five patients described an incomplete response to treatment (243, 360, 365, 356 and 261). Three (243, 360, 365) satisfied three criteria, while case 356 satisfied two (automatisms/unresponsiveness and treatment response, albeit partial) and case 261 satisfied one (clear-cut but incomplete treatment response). All had experienced frequent events of typical duration (<1 h), some occurring on waking. Three patients were in their sixties, two in their fifties. One patient, an overseas patient assessed in the UK but not included in the current series, described episodes of typical duration, occurring at roughly monthly intervals, often on waking, and gave a clear description of precipitation of episodes by exertion, a feature more often associated with TGA; some episodes were accompanied by olfactory hallucinations and video-telemetry confirmed the diagnosis of epilepsy. Thus, these atypical features generally occurred singly, in patients whose characteristics were otherwise typical for TEA, with no suggestion of distinct subgroups or likely alternative diagnoses.

Finally, in some patients, the clinical phenotype falls in a grey zone between ‘typical’ temporal lobe or limbic epilepsy, and TEA. For example, patients with focal seizures with impaired awareness sometimes exhibit a period of prominent postictal amnesia. There is also a group of patients with temporal lobe epilepsy who present with notable interictal memory disturbance of the kind associated with TEA, accompanied by subtle seizures, but who never have amnestic events of the kind required for a diagnosis of TEA. The term ‘Epileptic Amnesic Syndrome’ has been proposed to accommodate patients with temporal lobe epilepsy (TLE) accompanied by such interictal memory disturbance regardless of whether they do or do not also have amnestic seizures.^21^^,^^39–41^ The features of such borderline cases do not, however, call into question the existence of the core syndrome of TEA.

Thus, TEA is a distinctive epilepsy syndrome, with substantially consistent features across the two series of patients described from our centre and in recent reports from other centres. The relatively minor inconsistencies between our two series are likely to be explained by heightened awareness of the clinical features by the time we studied our second series. The existence of ‘atypical’ and ‘grey’ cases indicates that there are areas of overlap between TEA and other related epileptic conditions but does not invalidate the proposal that TEA is a distinctive epilepsy syndrome.

### The nature of the interictal memory disturbance in TEA

The majority of patients with TEA describe characteristic symptoms of interictal memory disturbance, in particular symptoms of ALF, AbA and TopA. These are often the most prominent and sometime the earliest symptoms of TEA.^6^^,^^9^^,^^21^^,^^42^ As only ALF and AbA have been studied in detail in the context of TEA we will focus on these here.

**Figure 4:**
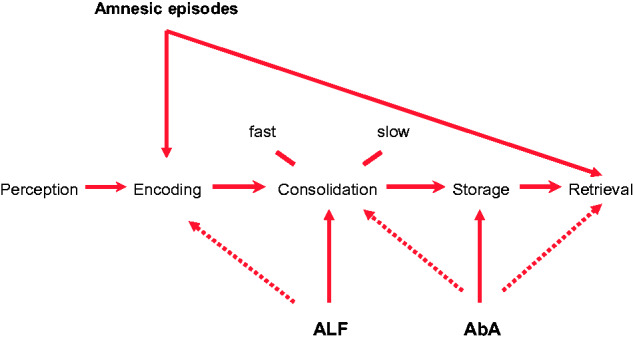
**Mechanisms of amnesia occurring in TEA.** The mechanisms of the three well-studied forms of amnesia occurring in TEA in relation to the key stages of memory processing: Episodes of ictal amnesia (‘TEA’) result from impairment of memory encoding, retrieval or, often, both; ALF is due to an impairment of consolidation processes, with a possible contribution from an encoding impairment; the AbA probably results from memory degradation or erasure, in the case of remote memories, but loss of access, impairing retrieval and ALF, affecting consolidation, can also play a part.

These phenomena are now established clinical entities with operational definitions. Some individuals with TEA—and other conditions—who perform within normal limits on standard measures of memory nevertheless have measurable evidence of ALF and/or AbA in the presence of corresponding symptoms. There is, however, continuing uncertainty about the pathophysiology of these phenomena, specifically regarding which phase of memory processing is perturbed (see Fig. 4) and whether the underlying cause is a disturbance of function or of structure.

In the case of ALF, the presence of memory impairment at extended intervals in patients with apparently normal learning, and intact early recall, suggests an impairment of memory consolidation. This interpretation is supported by examples of patients with impeccable performance on anterograde tests at standard intervals who nonetheless show marked accelerated long-term forgetting.^37^^,^^42–45^ Existing evidence indicates that ALF is first detectable within hours of learning,^46^ and that it occurs predominantly during wakefulness rather than sleep, perhaps hinting at an increased sensitivity to retroactive interference.^46^^,^^47^ However, there is also evidence that patients with TEA show early forgetting, over standard intervals, on recognition tests using visual materials,^48^^,^^49^ and some work in patients with TLE has suggested that ALF flows from an impairment of memory acquisition.^50^ Thus, it remains controversial whether ALF reflects a true impairment of memory consolidation or rather the increasing sensitivity of memory tests at longer intervals to impairments present from, or very close to, the point of memory acquisition. We have previously suggested that this may be, in part, a false dichotomy, as such impairments will often coexist and interact.^45^^,^^51^

There is also uncertainty over the relative importance of reversible functional factors, particularly ictal or interictal discharges versus structural factors in the causation of ALF. Studies—predominantly in patients with TLE—identifying a positive correlation between seizure frequency and ALF,^52–54^ interictal discharge frequency and ALF,^53^^,^^55^ and reduction of ALF by anticonvulsant treatment^56^^,^^57^ and epilepsy surgery^58^ argue for the importance of functional disturbance. The apparent reversibility of ALF in some cases of TEA also points to a modifiable cause.^59^ However, other studies have failed to identify such relationships.^42^^,^^60^^,^^61^ Moreover, ALF has recently been reported in patients with pre-symptomatic genetically determined Alzheimer’s disease^62^^,^^63^ and in children following head injury,^64^ suggesting that epileptiform brain activity is probably not required for its occurrence. Conversely, Butler et al^37^^,^^65^ found no correlation between volumes of limbic structures and the severity of ALF, arguing against a straightforward structural explanation of ALF in TEA. Thus, just as impairment of both early and later phases of memory processing are likely to contribute to ALF, so it seems likely that both functional and structural factors may be relevant, though the evidence in TEA somewhat favours the importance of functional disturbance. In a detailed single case study, the resolution of ALF on withdrawal of the inciting agent—high dose intrathecal baclofen—clearly demonstrated a reversible, pharmacological, cause.^45^

With respect to the AbA associated with TEA, detailed case studies indicate that TEA can erase or render inaccessible previously detailed autobiographical memories.^9^^,^^45^^,^^66^ This points to a disorder of storage, or possibly retrieval, although even rich retrieval cues failed to elicit recollection in these cases, arguing that in general storage is the more likely locus of pathology. We have, however, previously reported one patient who unexpectedly ‘recovered’ memories indicating that retrieval failure is, at least sometimes, the explanation for AbA in this condition.^67^ Finally, individuals with ALF would be expected to develop a degree of AbA over time for events occurring after the onset of ALF: The ‘disappearance’ of initially detailed memories, of the kind predicted by this hypothesis have been documented in the cases of CS^45^ and MB.^9^ Mosbah et al.^6^ reported an improvement in autobiographical recall for recent events following treatment, in keeping with this possibility; Savage et al.^68^ report a similar improvement in patient CS. Thus it is likely that AbA in TEA can reflect disorders of several stages of memory processing, including consolidation, storage and retrieval. Our working hypothesis is that the primary mechanisms of AbA in TEA, particularly in cases with temporally extensive memory loss, is degradation of stored engrams. One further possibility is worth considering, though there is at present no relevant evidence: That the AbA of TEA is due to a problem with reconsolidation.^69^ This could account for the close association between ALF and AbA.

Anecdotal evidence points to a role for epileptic activity in the genesis of TEA-related AbA. This idea receives some support from the significant negative correlation, reported above, between total number of seizures and the episodic memory score in the current (TIME2) patient series. The probable though controversial association between electroconvulsive therapy, which produces iatrogenic seizures and AbA,^70^ is also in keeping with the hypothesis that TEA-related AbA is at least partly the outcome of epileptic activity propagating through the autobiographical memory network, resetting the synaptic weights on which episodic autobiographical memories are likely to depend. Although there is evidence in other contexts that structural brain damage can produce AbA,^9^^,^^71^ to date there is no evidence of any correlation between volume loss in limbic structures and AbA in patients with TEA. Thus, the available evidence favours a role for epileptiform activity in the causation of AbA in TEA, with a possible, but so far unquantified, contribution from structural factors.

Cognitive deficits occurring in epilepsy are sometimes caused by drug treatment and by mood disturbance. However, given that both ALF and AbA can be detected in patients with TEA prior to treatment^6^ and that there is no evidence for an elevated rate of mood disturbance in the majority of patients^5^^,^^6^; this series), neither explanation is likely here.

In summary, ALF and AbA, both common interictal features of TEA, reflect disorders of several stages of memory processing, affecting acquisition and consolidation in the case of ALF, storage and retrieval in the case of AbA for remote memories (with a contribution from ALF in the case of Aba for post-onset memories). Current evidence favours a predominant role for neurophysiological factors in causing these phenomena in TEA, though structural factors are undoubtedly relevant in other contexts, and may also play some role in TEA.

### A model of TEA

**Figure 5:**
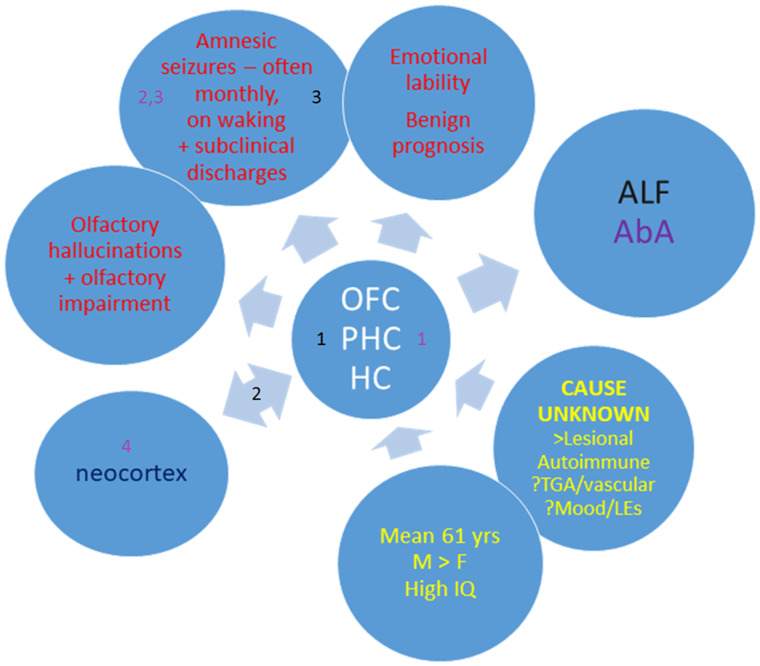
**A model of TEA. Yellow text summarizes demographic and aetiological factors;** the central circle highlights the limbic regions likely to contain the seizure focus; red text summarizes the key clinical features, which also include interictal memory disturbance, ALF (black) and AbA (white). The numbers refer to possible mechanisms for ALF and AbA (see main text for further discussion); the neocortex interacts with limbic regions in memory processing. HC = hippocampus; LEs = life events; OFC = orbitofrontal cortex; PHC = parahippocampal cortices (including perirhinal cortex).

The aim of this section is to summarize current knowledge of TEA, identify unanswered questions and propose testable hypotheses regarding the underling mechanisms (Fig. 5).

#### Aetiology

The majority of cases of TEA are of unknown aetiology, but its typical occurrence in middle aged people, with a probable male predominance, points to an age-related susceptibility and the possible relevance of hormonal factors. Although TGA is unlikely to result from epilepsy in the majority of cases, the similarity in the ages of patients affected by TGA and TEA suggests that common age-related factors underlie both conditions. Rare symptomatic (lesional) cases of TEA are described and discussed further below. We have recently reported a case of TEA occurring secondary to NMDAR antibody mediated encephalitis.^68^ Transient amnesia has been described as a seizure type among patients with Alzheimer’s disease, but we have not seen the typical syndrome of TEA occurring in the context of Alzheimer’s disease.^72^^,^^73^ The significance of the high IQ of the patients in our series is uncertain, but we suspect it reflects the need for an articulate description of confusing symptoms in the diagnosis of TEA. The possibility that vascular risk factors may predispose to TEA was raised tentatively in a previous case control study, with associations—on the borderline of significance after correction for multiple comparisons—between TEA and cardiac arrhythmia, valve disease and arterial aneurysm.^74^ Some patients with TEA have an initial episode closely resembling TGA, posing the question of whether TGA can sometimes lead to TEA. Mosbah et al.^6^ noted a high frequency of depression and adverse life events preceding the onset of TEA in their cases: We have also encountered individual cases in which mood disorder and life events are plausible triggers, but these relationships require further systematic study before firm conclusions can be drawn.

#### Seizure source

Manual and automated measurement of brain structures in TEA has revealed mild atrophy of limbic regions, namely bilateral straight gyrus, medial orbital gyrus, hippocampus and right perirhinal cortex.^37^^,^^65^ In the minority of cases with a likely structural cause for TEA, the causative lesion usually lies within or close to this group of regions. In a single case study with radiological localization, an exacerbation of TEA was associated with swelling and high signal in the left hippocampus with hypermetabolism in the same region on a fluorodeoxyglucose PET scan which resolved with symptom improvement.^37^ This patient went on to develop left hippocampal atrophy. In cases with epileptiform interictal EEGs, or ictal recordings, the discharges are temporal or fronto-temporal, in keeping with the localization suggested by brain imaging. Therefore, TEA can be regarded as a form of temporal lobe epilepsy. However, as some cases may originate in extra-temporal (e.g. inferior-orbital) areas, it may be more correct to describe TEA as a subtype of limbic epilepsy.

#### Seizure characteristics

The amnesic episodes in TEA, which typically last around half an hour, are unusually prolonged for epileptic seizures: We discuss their mechanism further below. Their occurrence on waking is in keeping with a medial temporal seizure source.^75^^,^^76^ Sleep EEG recordings can be useful in diagnosis.^6^ The high frequency of monthly episodes is a striking and puzzling feature, hinting at some underlying process with a similar time course involving limbic cortices, but cyclical epilepsy is described in other contexts.^77–79^ Olfactory hallucinations, sometimes prolonged and both subjective and objective alterations in olfaction, are common in TEA, at least in our experience, and can provide a diagnostic clue. Amnesia occurs as the sole ictal manifestation in around one quarter of cases of TEA; other manifestations include olfactory hallucinations, brief loss of awareness, automatisms and, rarely, tonic–clonic seizures.

#### Seizure mechanism

Surface EEG recording during an amnesic attack was performed in nine literature cases and one TIME1 case.^4^ All recordings showed seizure activity, which in 8/10 cases involved both temporal lobes and in the others remained unilateral (one left sided and one right sided). Amnesia was observed as an ictal phenomenon in six cases and as postictal in four cases. In some ictal cases the underlying seizure activity was prolonged,^80^ accounting for the correspondingly prolonged amnesic state This suggests that the amnesia occurring in episodes of TEA can occur both as true ictal manifestations and as postictal phenomenon, a ‘Todd’s paresis’ of memory. The precise temporal relationship between seizure onset and offset and the associated amnesia is, however, uncertain in the majority of cases of TEA.

#### Emotional lability

We noted a characteristic form of emotional lability in 18% of our first series of patients, and in 40% of the current series, a result, we suspect, of greater awareness of this feature. This typically involves a heightened emotional reactivity to poignant but relatively minor triggers, such as a story or tune on radio or TV, or a social encounter, often leading to unexpected tearfulness. A strikingly similar phenomenon has been described recently among patients with limbic encephalitis.^81^

#### Interictal memory disturbance

Figure 5 indicates potential mechanisms for ALF and AbA. For ALF, ‘1’ refers to the possibility that a focal pathology or disturbance in function of limbic structures, most likely the hippocampus, disrupts memory acquisition and/or consolidation. The pathology could be integral to the underlying cause of the epilepsy, a structural result of the epilepsy or reflect an adaptation to the epilepsy: Taking our observation that an intrathecal-administered Gamma aminobutyric acid (GABA) receptor B agonist, Baclofen, can cause ALF,^45^ together with evidence that experimental epilepsy can induce compensatory inhibitory mechanisms,^82^ we hypothesize that excessive inhibition within the medial temporal lobe memory system may be a mechanism of ALF.^83^ Second (‘2’), disruption of the normal dialogue between the medial temporal lobe and the neocortex, required for the consolidation of recently acquired memories could play a role.^51^ Finally (‘3’)—and not to the exclusion of 1 and/or 2—ictal and interictal discharges may disrupt memory processing.

In the case of AbA, structural pathology (‘1’) in the medial temporal lobe, perhaps detectable using high field MRI imaging,^71^^,^^84^^,^^85^ could underlie the depletion of autobiographical memories that occurs in TEA, in keeping with the ‘multiple (hippocampal) trace’ model of remote memory.^86^ Ictal or interictal discharges may delete (‘2’) or render inaccessible (‘3’) engrams in the medial temporal lobe (‘1’) or neocortex (‘4’).

## Conclusion

The growing world literature on TEA included 94 cases at the time of our previous review in 2008, among them 50 from our first series; in the current paper we report a further 65 patients studied at our centre and 114 cases from elsewhere reported since 2008. This now substantial patient cohort indicates that TEA is a distinctive form of late-onset limbic epilepsy. It gives rise to recurrent episodes of transient amnesia, typically lasting for around 30 min, often on waking, frequently occurring at intervals of around one month. The predominantly ‘memory-related’ presentation of TEA often leads to referral to memory clinics or for psychiatric assessment; diagnosis is frequently mistaken or delayed. Olfactory hallucinations are a common accompaniment and useful diagnostic clue. There is, in several series, an unexplained male predominance. Interictal memory impairment, specifically ALF and AbA occur in the majority of patients, sometimes associated with a distinctive form of emotional lability. The condition is most often of unknown aetiology, and such cases have a benign prognosis. TEA occasionally occurs as a result of structural pathology and as a manifestation of auto-immune epilepsy. The aetiology of most cases of the condition, the monthly occurrence of seizures in some patients and the mechanisms and interrelationships of the interictal features—amnestic and affective—all warrant further study. The current report establishes TEA as an important, treatable cause of memory loss in older people, often mistaken for dementia, cerebrovascular disease and functional amnesia.

## Supplementary material

Supplementary material is available at *Brain Communications* online.

## Funding

John Baker received funding from the Alzheimer’s Society (Alzheimer's Society Doctoral Training Centre in Dementia Research at the University of Exeter; grant reference 231).

Christopher Butler received funding from the Medical Research Council (Clinician Scientist Fellowship MR/K010395/1).

Adam Zeman received funding from the Dunhill Medical Trust (grant number R322/1113).

## Conflicts of interest

The authors report no conflicts of interest.

## Supplementary Material

fcab038_Supplementary_Data

## Abbreviations

AbA: Autobiographical amnesia
ALF: Accelerated long-term forgetting
ECT: electroconvulsive therapy
HADS: Hospital anxiety and depression scale
HC: Hippocampus
LEs: Life events
MAMI: Modified autobiographical memory interview
MTL: mesial temporal lobe
OFC: Orbitofrontal cortex
PHC: Parahippocampal cortices (including perirhinal cortex)
TEA: Transient epileptic amnesia
TGA: transient global amnesia
TIME1: The impairment of memory in epilepsy study: phase 1
TIME2: The impairment of memory in epilepsy study: phase 2
TopA: Topographical amnesia
