# Supplementary material for: The syndrome of transient epileptic amnesia: a combined series of 115 cases and literature review
Source: Brain Commun. 2021 Mar 13;3(2):fcab038. doi: 10.1093/braincomms/fcab038 (PMC8047097; doi:10.1093/braincomms/fcab038)

Supplementary Table 1: Characteristics of TIME2 patient cohort ("partial" indicates a >50% reduction in seizure frequency)

Study ID	Sex	Age at Onset (yr)	Total number of attacks	first to last attacks (mo)	Duration of attacks	Amnesia on Waking	Number of Criteria Met	EEG	Other Features sometimes Present	Treatment Response
191	M	66	10	21	5-15 minutes	Y	3	Epil	Autom, Unresp	Com
205	F	57	12	22	15-30 minutes	Y	3	Epil	Olf hall, Autom, Unresp	Com
220	M	60	36	36	15-30 minutes	Y	3	Epil	Autom, Unresp	Com
222	M	62	50	11	5-15 minutes	Y	3	Epil	Autom, Unresp	Com
235	M	61	50	64	1-5 minutes	Y	3	Epil	Olf hall, Autom, Unresp	Com
236	M	66	15	22	30 minutes to 1 hour	Y	3	Epil	Olf hall	Com
243	F	64	10	24	5-15 minutes	Y	3	Epil	Olf hall	Partial
260	M	44	175	26	30 minutes to 1 hour	Y	3	Epil	Olf hall, Autom	Com
282	F	58	200	4	5-15 minutes	Y	3	Epil	Autom, Unresp	Com
305	F	26	10		15-30 minutes	Y	3	Epil	Unresp	Com
336	M	44	50	8	5-15 minutes	Y	3	Epil	Olf hall, Autom, Unresp	Com
343	M	54	12	6	1-5 minutes	Y	3	Epil	Olf hall, Autom, Unresp	Com
358	M	47	50	76	30 minutes to 1 hour	Y	3	Epil	Autom	Com
360	M	67	20	84	30 minutes to 1 hour	Y	3	Epil	Autom	Partial
361	F	55	5	4	15-30 minutes	Y	3	Epil	Olf hall	Com
365	M	53	40	108	1-5 minutes	Y	3	Epil	Olf hall, Autom, Unresp	Partial
367	M	60	37	18	15-30 minutes	Y	3	Epil	Olf hall, Autom	Com
368	F	66	10	12	30 minutes to 1 hour	Y	3	Epil	Olf hall, Unresp	Com
375	M	72	172	22	1-5 minutes	Y	3	Epil	Autom, Unresp	Com
393	M	69	20	16	1-2 hours	Y	3	Epil	Autom	Com
195	M	59	4	37	1-2 hours	Y	2	Non-spec	Olf hall, Autom, Unresp	Com
213	F	55	24	24	1-5 minutes	N	2	Epil		Com

223	M	72	12	37	1-2 hours	Y	2	Normal	Autom, Unresp	Com
226	M	66	6	7	15-30 minutes	Y	2	Non-spec	Olf hall	Com
229	F	76	4	3	1- 5 minutes	N	2	Normal	Olf hall, Unresp	Com
232	M	66	19	36	1 -2 hours	Y	2	Normal	Autom, Unresp	Com
238	M	51	16	62	30 minutes to 1 hour	Y	2	Normal	Olf hall, Autom, Unresp	Com
241	M	52	14	7	2-24 hours	Y	2	Non-spec	Unresp	Com
251	M	66	2	23	1-2 hours	Y	2	Non-spec	Unresp	Com
254	M	66	70	16	5-15 minutes	Y	2	Normal	Autom, Unresp	Com
272	M	62	100	67	1-5 minutes	Y	2	Non-spec	Autom, Unresp	Com
277	M	63	4	14	5-15 minutes	Y	2	Normal	Olf hall	Com
288	M	69	6	86	1-2 hours	Y	2	Normal	unresp	Partial
292	M	77	30	43	15-30 minutes	N	2	Non-spec	Unresp	Com
317	F	76	50	23	1-2 hours	N	2	Normal	Olf hall, Unresp	Com
325	F	66	4	6	1-5 minutes	Y	2	Non-spec	Unresp	Com
346	F	56	15	44	2-24 hours	Y	2	Non-spec	Olf hall	Com
349	M	43	20	26	1-5 minutes	Y	2	Normal	Unresp	Com
351	M	65	24	120	5-15 minutes	Y	2	Not done	Olf hall, Autom, Unresp	Com
352	M	54	12	12	15-30 minutes	Y	2	Non-spec	Olf hall	Com
355	M	56	10	8	15-30 minutes	Y	2	Normal	Unresp	Com
356	M	67	70	70	15-30 minutes	Y	2	Normal	Autom, Unresp	Partial
359	M	65	20	33	5-15 minutes	Y	2	Normal	Olf hall, Unresp	Com
362	M	52	50	2	<1 minute	Y	2	Non-spec	Olf hall, Unresp	Com
371	M	71	6	1	30 minutes - 1 hour	Y	2	Non-spec	Olf hall	Com
373	F	70	12	56	15 -30 minutes	Y	2	Epil		Com
374	M	66	4	6	1-2 hours	Y	2	Norm	Autom	Com
378	M	65	14	45	15-30 minutes	Y	2	Normal	Olf hall, Autom, Unresp	Com
379	M	69	8	12	30 minutes to 1 hour	Y	2	Non-spec	Olf hall, Unresp	Com

380	M	58	6	5	5 - 15 minutes	Y	2	Non-spec	Olf hall, Autom	Com
383	M	59	13	48	2-24 hours	N	2	Normal	Olf hall	Com
388	M	67			1-2 hours	Y	2	Non-spec	Olf hall, Autom	Com
394	M	72	9	10	15-30 minutes	Y	2	Not done	Autom, Unresp	Com
396	M	73	8	8	1-2 hours	Y	2	Not done	Olf hall, Autom	Com
193	M	71	15	11	1-2 hours	Y	1	Non-spec		Com
207	M	63	4	21	15-30 minutes	Y	1	Not done		Com
217	M	72	2	1	15-30 minutes	Y	1	Non-spec		Com
218	F	42	100	84	30 minutes to 1 hour	Y	1	Normal		Com
257	M	57	50	53	2-24 hours	Y	1	Normal		Com
261	M	59	50	52	5-15 minutes	Y	1	Normal		Partial
322	M	66	2	93	15-30 minutes	Y	1	Non-spec		Com
340	F	39	5	6	1-2 hours	Y	1	Normal		Com
341	M	75	5	7	30-60 mins	Y	1	Normal		Com
363	M	71	17	18	30 minutes to 1 hour	Y	1	Normal		Com
376	M	55	6	10	15-30 minutes	Y	1	Normal		Com

Supplementary Figure 1: Annual frequency (a) and duration of epileptic seizures (b) in TIME2 group

(a) (b)

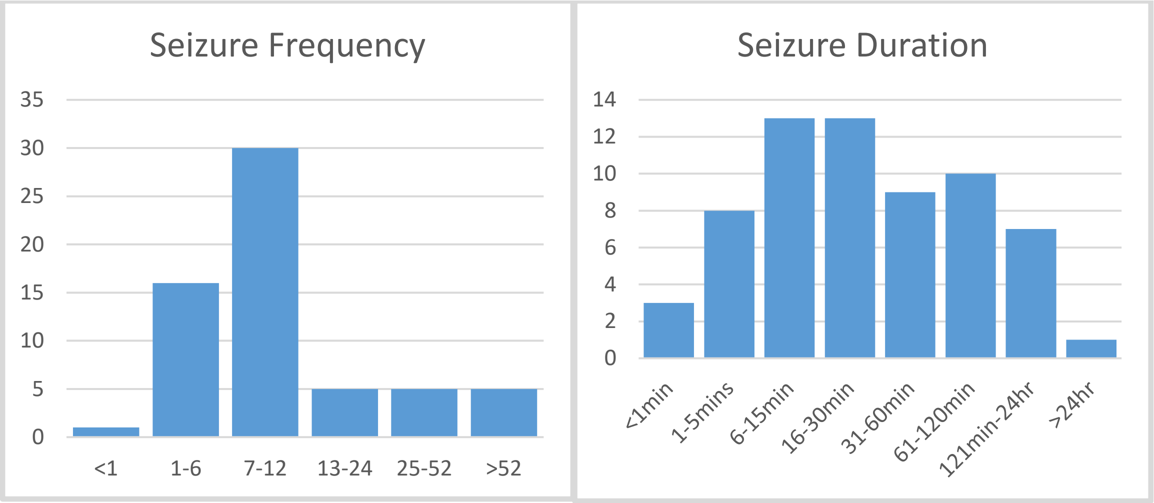

Supplementary Figure 2: EEG flow-chart for TIME2 cohort (in cases where multiple EEGs were performed, the final test listed provided the epileptiform result)

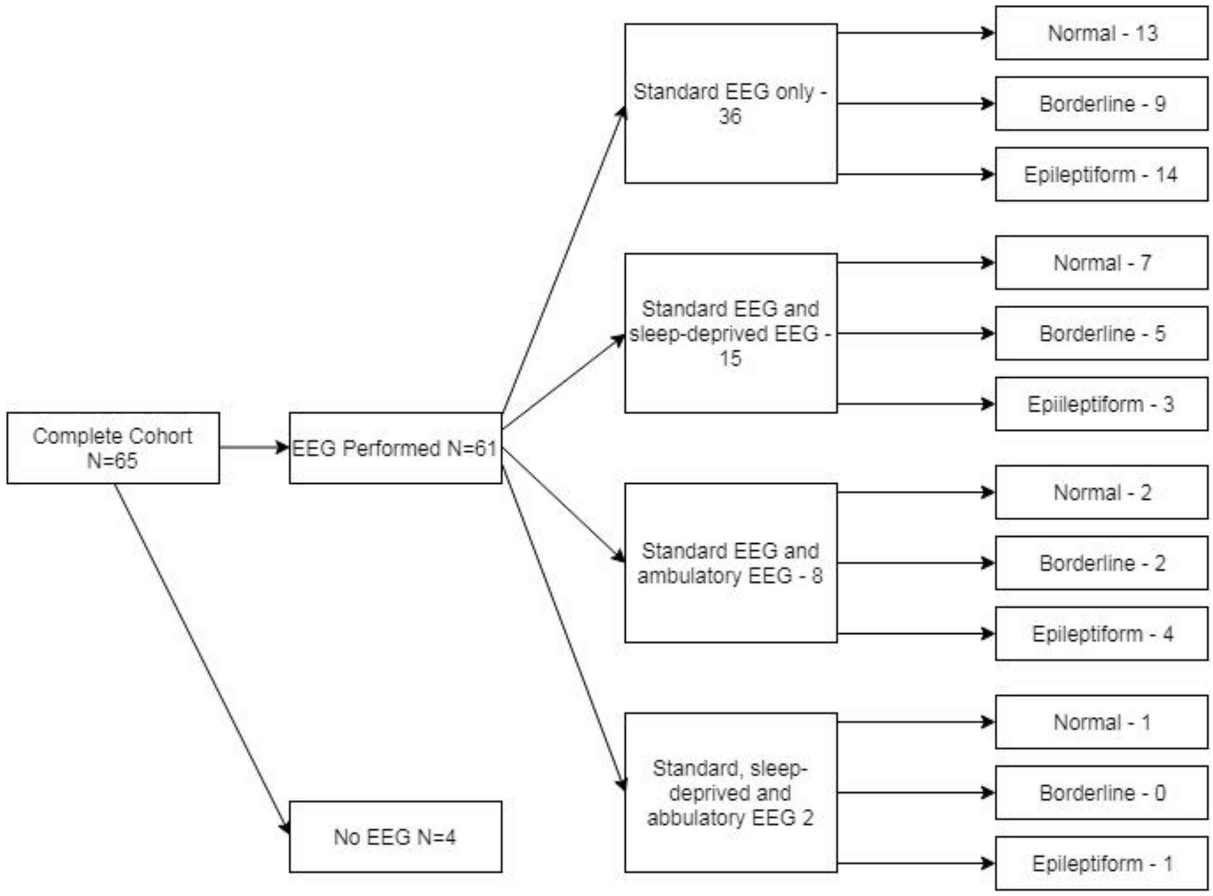

Supplementary Figure 3: Flow diagram demonstrating study selection process for literature review

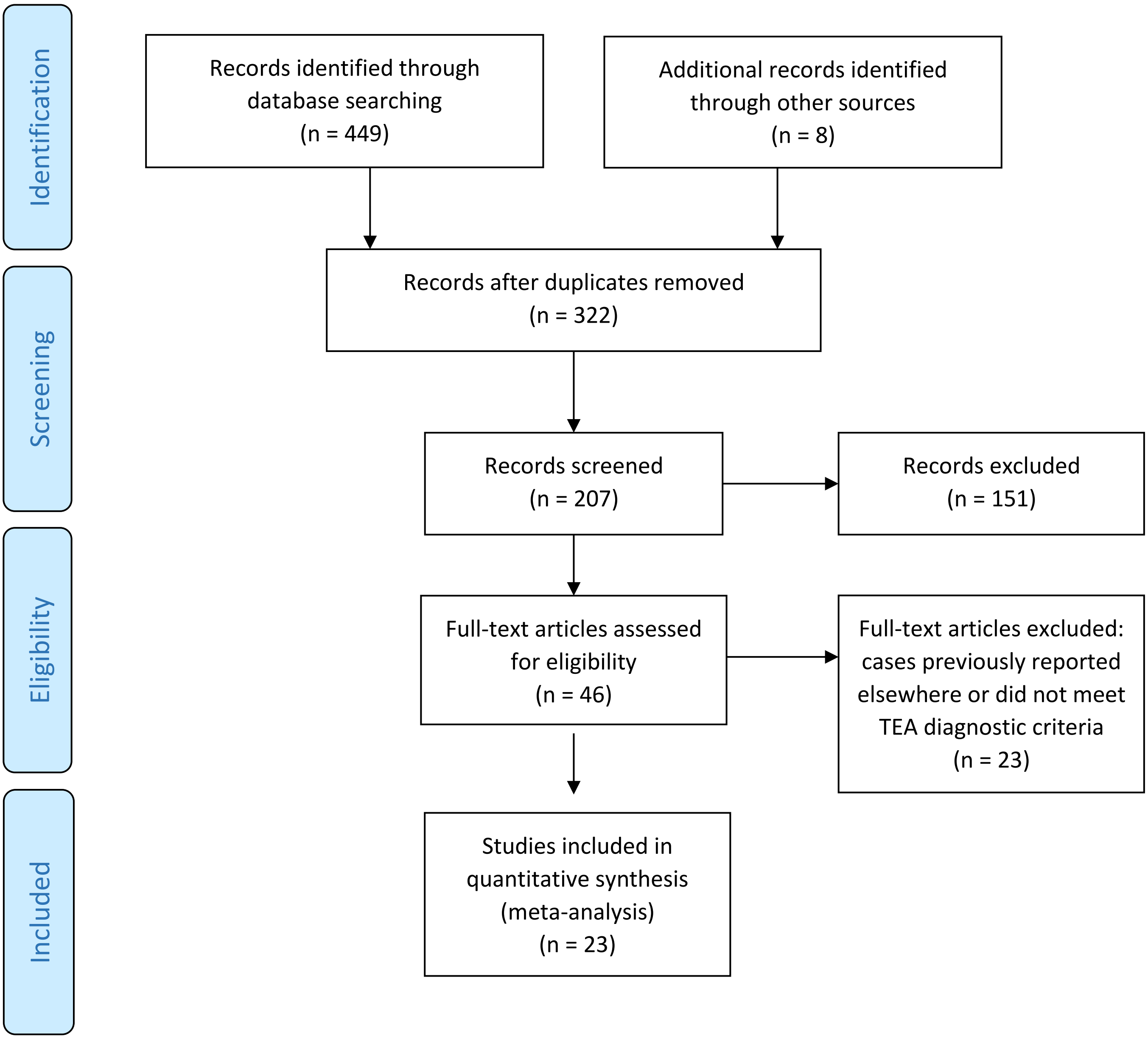

Supplement: fcab038_Supplementary_Data [file fcab038_supplementary_data.pdf]
